# High Th17:Treg ratio may predict complete response to HDIL-2 in the setting of melanoma

**DOI:** 10.1186/2051-1426-3-S2-P297

**Published:** 2015-11-04

**Authors:** Maggie L Diller, Ford Mandy, Keith A Delman, David H Lawson

**Affiliations:** 1 Department of General Surgery, Emory University, Atlanta, GA, USA; 2 Emory University Transplant Center, Atlanta, GA, USA; 3 Emory University Winship Cancer Institute, Atlanta, GA, USA

## Introduction

In vitro and in vivo experiments have demonstrated a potent yet opposite effect of IL-2 on both regulatory T cells (T_REG_) and IL-17+CD45RA-CD4+ T cells (Th17). T_REG_ cells have been implicated as an important immunoregulator enhancing tumor growth whereas Th17 cells may mediate tumor destruction. This study compares the effect of high-dose IL-2 (HDIL-2) on both the TREG and Th17 compartments in responders and non-responders.

## Methods

Peripheral blood was collected at baseline and at 24, 48, 72, and 96 hours post-treatment from 6 patients undergoing HDIL-2 therapy under an IRB approved protocol. No patients enrolled received anti-PD-1 or anti-CTLA4 therapies. PBMCs were isolated and underwent intracellular cytokine and extracellular receptor staining for flow cytometry. Statistical analysis was performed using paired student's t tests via Prism 6.0e software.

## Results

**Figure 1:**
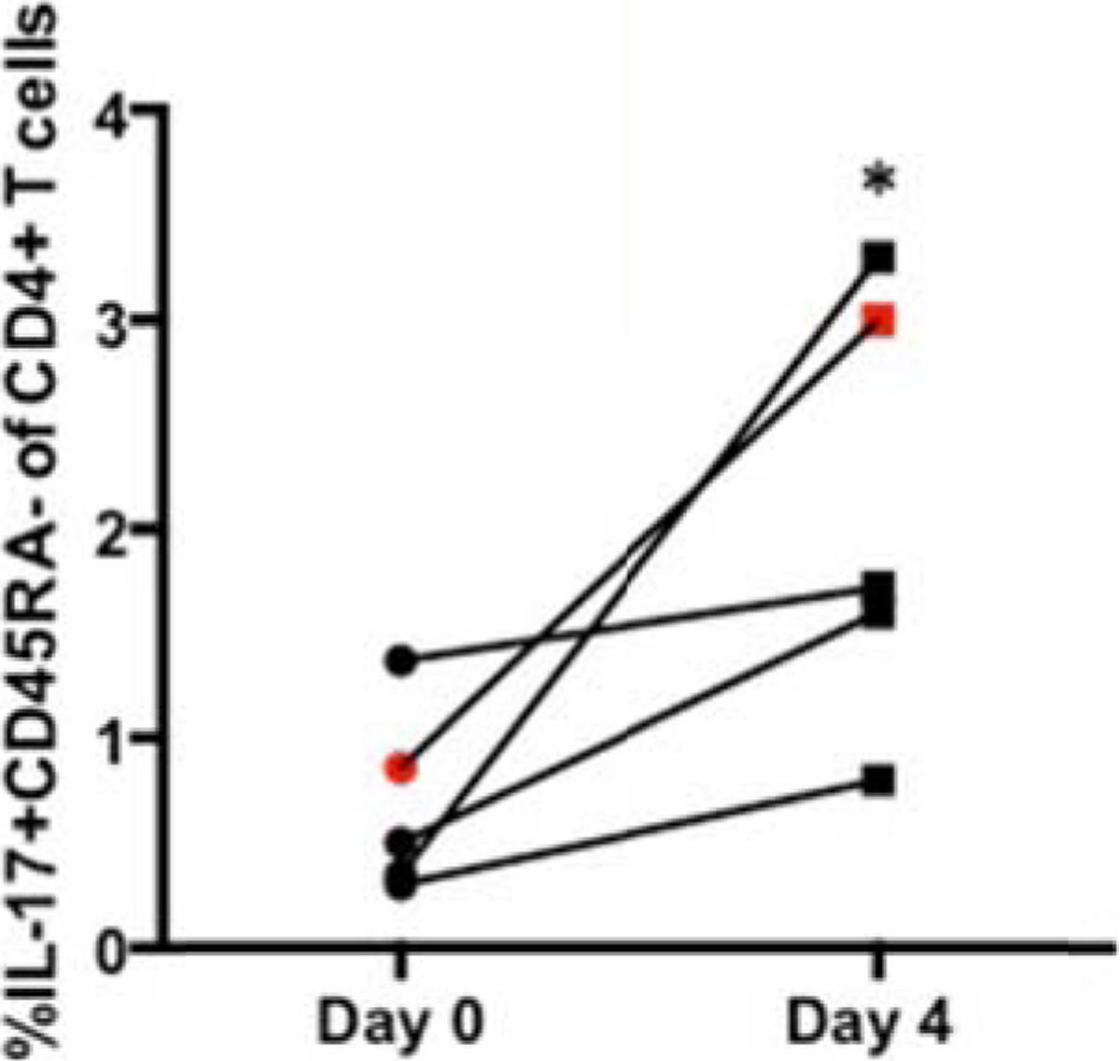


**Figure 2:**
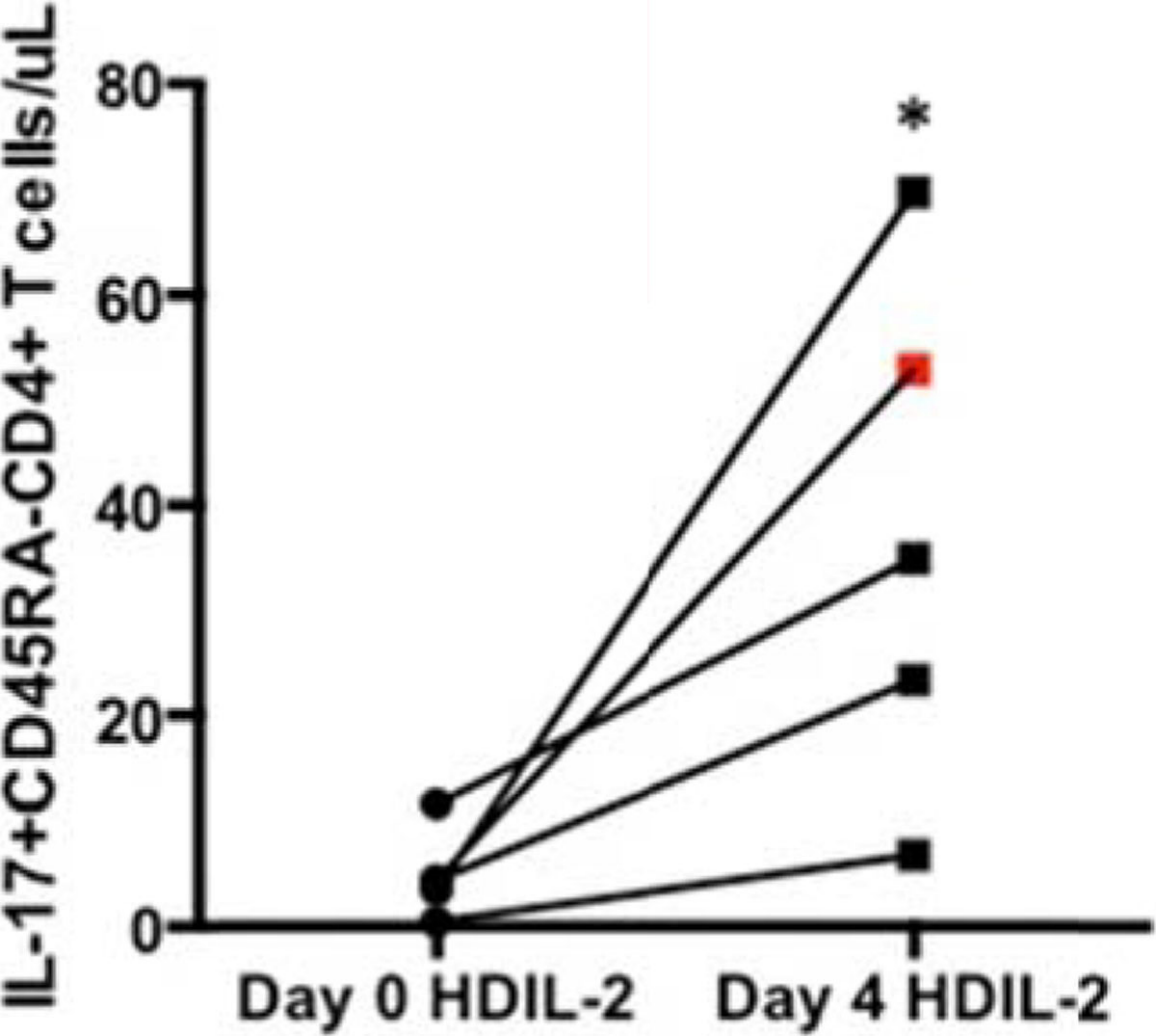


**Figure 3:**
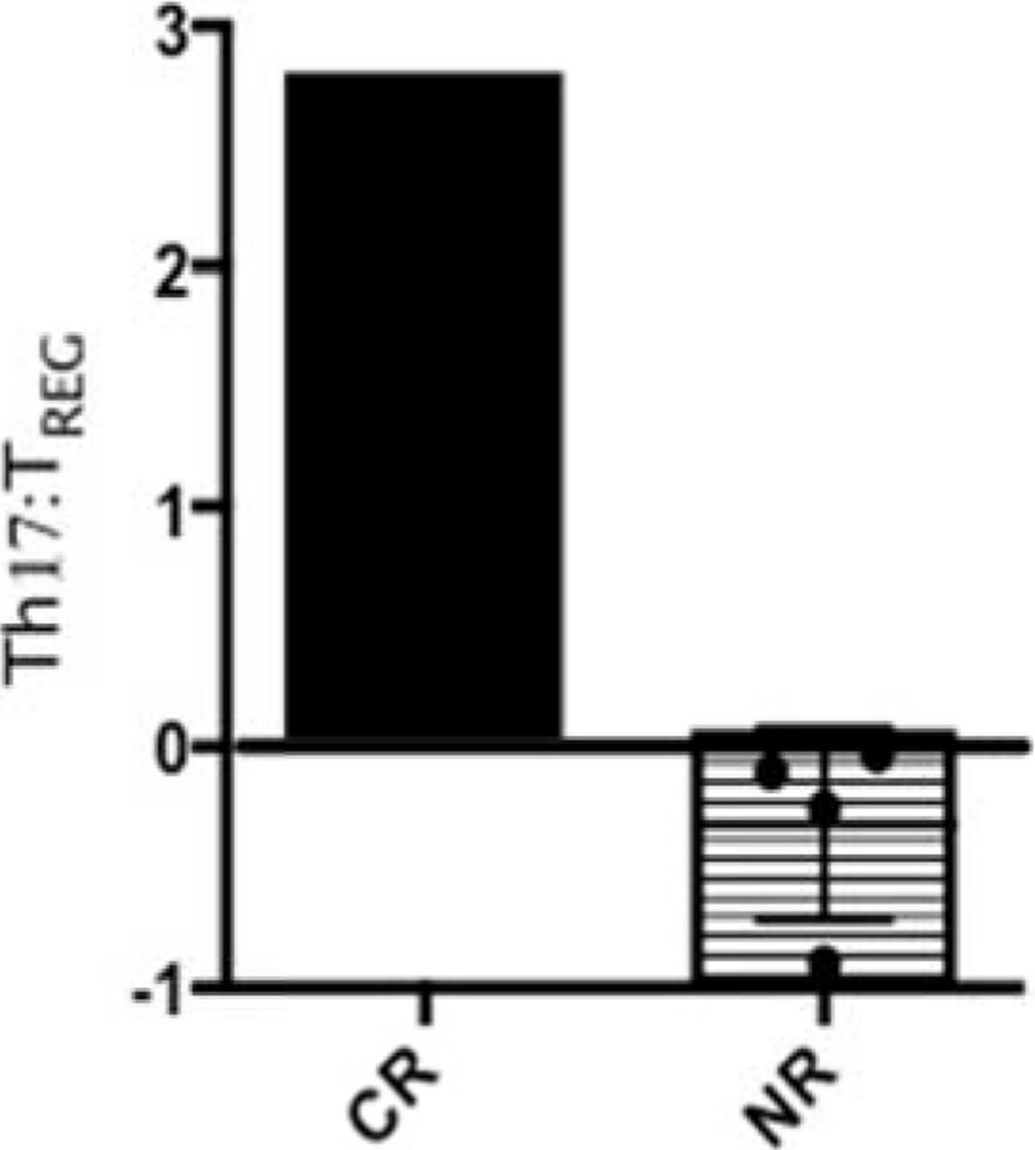


5 of 6 patients clinically progressed on HDIL-2 therapy (non-responders, NR), and these patients demonstrated an increase in the frequency of CD25+FoxP3+CD4+ T cells (T_REG_) on day 4 of treatment (4% +/- 1% on day 0 to 14% +/- 6% on day 4, p value = 0.06). A single patient responded to HDIL-2 therapy (complete responder, CR) and demonstrated a decrease in the frequency of T_REG_ cells on day 4 of treatment (9% on day 0 to 7% on day 4). HDIL-2 increased the frequency of IL-17+CD45RA-CD4+ T cells (Th17) on day 4 of therapy in all patients analyzed (0.7% +/- 0.4% on day 0 versus 2% +/- 1% on day 4, p value=0.04; Figure 1). Absolute numbers of Th17 cells also demonstrated a statistically significant increase on day 4 of therapy (5 +/- 1 cell/µL on day 0 versus 38 +/- 24 cells/µL on day 4, p value=0.04, Figure 2). Subsequent analyses demonstrated a negative Th17:T_REG_ ratio on day 4 of HDIL-2 treatment in all non-responders. Importantly, the complete responder demonstrated a positive Th17:T_REG_ ratio on day 4 of treatment (Figure 3). The observed difference in cytokine production appeared to be specific to IL-17 as there was no statistically significant change in frequency or total numbers of IFN-g+ or IL-2+CD4+ and CD8+ T cells.

## Conclusion

Our results suggest a distinct immunophenotype indicative of response to HDIL-2. Analysis of peripheral T_REG_ and Th17 cell frequencies early in the course of HDIL-2 therapy may help identify those patients who would benefit from subsequent cycles.

